# The Influence of Colored Rays on Living Organisms; Tempora Mutantur

**Published:** 1872-12-15

**Authors:** 


The Influence of Colored Rays on Living Organisms. — Those of our readers who have given credence to the newspaper reports touching the remarkable growth—stimulating powers of the blue rays of light will find material for reflection in the following statements of Baudrimont:

"Noxious Influence of Certain Colors on Vegetation. — The communication of M. Poey, of Havana, relative to the influence of violet light on the well-being of plants and animals will be remembered by the reader. M. Baudrimont reports to the same Academy of Sciences that, since 1858, he had pursued investigations similar to those of M. Poey. He had obtained directly opposite results. All colors were unfavorable to vegetation, and none had proved more so than the violet. All the plants subjected to that light were the first to die. The most noxious color after violet was green. Blue, situated between these two, in an optical point of view, did not give such baneful results."—*La France Medicale.*

Tempora Mutantur.—*The sentiment of Parisian women in reference to abortion one hundred years ago.*— We translate from the notes of F. M. LeMoine, the editor of the French edition of that excellent work of John Burton, of York, on Midwifery, the following sentences, published in 1771. They furnish a striking contrast with the sentiment of the women of our day, and serve to indicate how far a people may degenerate in the comparatively short period of one hundred years:

"It cannot be too frequently enjoined upon pregnant women that they carry in their wombs, even in the first days of conception, a living and perfectly organized being. *Not that I fear* that they would undertake the destruction of the fruits of conception by the abominable methods mentioned by Mauriceau, for I believe if there exist to-day any mothers so unnatural as to commit so enormous a crime, their number must be very few, but I desire to inform them of the truth so that they may shun certain kinds of exercise, which are frequent causes of abortion. I know that accoucheurs have sometimes a motive in giving woman a contrary idea. A woman has a miscarriage, the first sentiment she experiences is one of regret and sorrow. She reproaches herself bitterly with not having been able to carry to full term the fruit of conception. Meanwhile, to console her, she is told that she was not yet sufficiently advanced in pregnancy that the embryo should have possessed life, or, that it was only a false conception. It is certainly pardonable to deceive her for the time being, for her acute grief in her then condition might have proved dangerous; but when she has recovered, the truth should be told to her. It must be acknowledged to the honor of women, even of those most given up to pleasures, that upon the occurrence of miscarriages they repent of any imprudence which they may have committed, and deploring the unfortunate results, promise in another pregnancy to conduct themselves with more caution."

